# Trends of Mortality due to Septicemia in Greece: An 8-Year Analysis

**DOI:** 10.1371/journal.pone.0067621

**Published:** 2013-07-02

**Authors:** Matthew E. Falagas, Ioanna P. Korbila, Anastasios Kapaskelis, Kyriaki Manousou, Lili Leontiou, Giannoula S. Tansarli

**Affiliations:** 1 Alfa Institute of Biomedical Sciences (AIBS), Athens, Greece; 2 Department of Internal Medicine - Infectious Diseases, Mitera Hospital, Hygeia Group, Athens, Greece; 3 Department of Applied Mathematics and Physics, National Technical University of Athens, Athens, Greece; 4 Department of Medicine, Tufts University School of Medicine, Boston, Massachusetts, United States of America; Amphia Ziekenhuis, Netherlands

## Abstract

**Background:**

Infectious diseases are among the major causes of death worldwide. We evaluated the trends of mortality due to septicemia in Greece and compared it with mortality due to other infections.

**Methods:**

Data on mortality stratified by cause of death during 2003–2010 was obtained from the Hellenic Statistical Authority. Deaths caused by infectious diseases were grouped by site of infection and analyzed using SPSS 17.0 software.

**Results:**

45,451 deaths due to infections were recorded in Greece during the 8-year period of time, among which 12.2% were due to septicemia, 69.7% pneumonia, 1.5% pulmonary tuberculosis, 0.2% influenza, 0.5% other infections of the respiratory tract, 7.9% intra-abdominal infections (IAIs), 2.5% urinary tract infections (UTIs), 2.2% endocarditis or pericarditis or myocarditis, 1.6% hepatitis, 1% infections of the central nervous system, and 0.7% other infections. A percentage of 99.4% of deaths due to septicemia were caused by bacteria that were not reported on the death certificate (noted as indeterminate septicemia). More deaths due to indeterminate septicemia were observed during 2007–2010 compared to 2003–2006 (3,558 versus 1,966; p<0.05).

**Conclusion:**

Despite the limitations related to the quality of death certificates, this study shows that the mortality rate due to septicemia has almost doubled after 2007 in Greece. Proportionally, septicemia accounted for a greater increase in the mortality rate within the infectious causes of death for the same period of time. The emergence of resistance could partially explain this alarming phenomenon. Therefore, stricter infection control measures should be urgently applied in all Greek healthcare facilities.

## Introduction

Infections are ranked among the top causes of death worldwide. [1] Severe infection can be complicated with sepsis and eventually with multiple organ dysfunction syndrome (MODS), which accounts for high mortality rates. [2] Risk factors associated with the development of sepsis include the presence of bacteraemia, of community-acquired pneumonia, the admission in the intensive care unit (ICU), the advanced age, and the impairment of immunologic responses. [1], [3], [4] Sepsis is most commonly caused by bacteria and to a less extent by fungi.

Infection caused by nosocomial pathogens is associated with higher mortality than the one by community-acquired pathogens, when complicated with sepsis. [5] Due to the development of adapting mechanisms to antimicrobials by the bacteria, accompanied with the difficulty in implementation of strict infection control measures, the prevalence of nosocomial multidrug-resistant bacteria imposes an increasing problem in the healthcare facilities. Greece is top-ranking among the countries facing a serious problem with the increase of multidrug-resistant nosocomial bacteria, especially the Gram-negative ones. However, it has not been studied yet the magnitude of the effect of the increased prevalence of Gram-negative bacteria on mortality.

In this context, we evaluated contemporary data with regard to the trends of mortality due to septicemia in Greece and compare with those due to other infections.

## Methods

### Data Collection and Analysis

Data on mortality, stratified by cause of death, was obtained from the Hellenic Statistical Authority. All the data was completely anonymous. The study protocol was approved by the Ethics Committee of the Alfa Institute of Biomedical Sciences.

All infectious causes of death during an 8-year period of time were grouped into distinct categories according to the site of infection: septicemia, endocarditis or pericarditis or myocarditis, respiratory tract infections (RTIs) including pneumonia, tuberculosis (TB), influenza or other infections of the respiratory tract, urinary tract infections (UTIs), intra-abdominal infections (IAIs), infections of the central nervous system (CNS), and hepatitis. Infections that could not be stratified into one of the aforementioned categories were grouped as “other infections”. The study period was divided into two quadrennials (2003–2006, 2007–2010) and a comparison between deaths of these two periods was performed for each site of infection separately.

### Definitions and Outcomes

Septicemia was defined as the presence of sepsis with confirmed or inderterminate bacteraemia. Inderterminate septicemia was defined as the one caused by bacteria that were not reported on the death certificate. The endpoint of the study was the trends of mortality due to septicemia as well as other infections in Greece during the 8-year period of time (2003–2010).

### Statistical Analysis

SPSS 17.0 software (SPSS Inc., Chicago, IL, USA) was used for all statistical analyses. Comparisons of continuous variables were performed with Student’s t-test or the Mann-Whitney U test (for normally or non-normally distributed variables, respectively). A p-value of <0.05 was considered to denote statistical significance.

## Results

**Figure 1:**
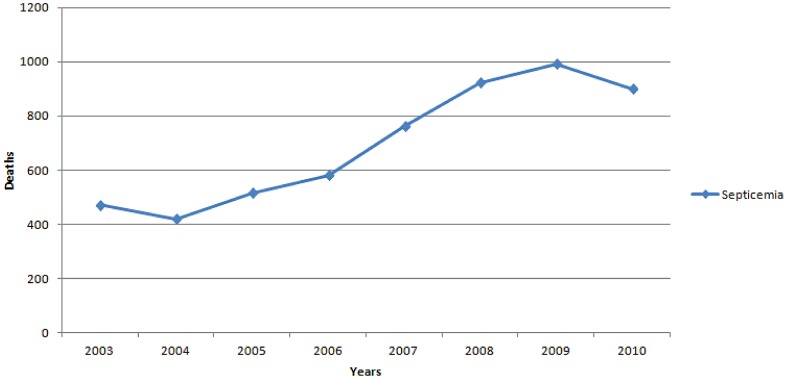
Deaths due to septicemia by year in Greece during 2003–2010.

Infectious diseases were the cause of 45,451 deaths during the period of 2003 to 2010 in Greece. In detail, 4,659 deaths due to infectious causes were recorded in 2003, 4,738 in 2004, 5,376 in 2005, 5,136 in 2006, 6,256 in 2007, 6,334 in 2008, 6,663 in 2009 and 6,289 in 2010. Among those, septicemia accounted for 5,556 (12.2%) deaths. *Staphylococcus* spp was the causative pathogen in 26 deaths due to septicemia (0.5%), *Streptococcus* spp in 6 (0.1%), while there were no deaths due to septicemia caused by anaerobes. Consequently, 99.4% (5,524) deaths due to septicemia had an undefined cause (noted as indeterminate septicemia). There were more deaths due to indeterminate septicemia during 2007–2010 than 2003–2006 (3,558 versus 1,966, 44.7% increase, p<0.05). The temporal trend of mortality due to septicemia during the 8-year period of time is depicted in Figure 1.

**Figure 2:**
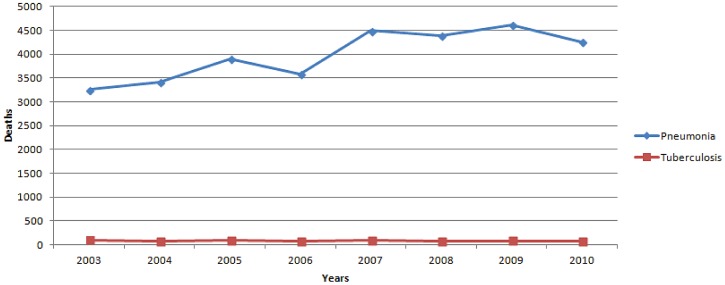
Deaths due to pneumonia and pulmonary tuberculosis by year in Greece during 2003–2010.

**Figure 3:**
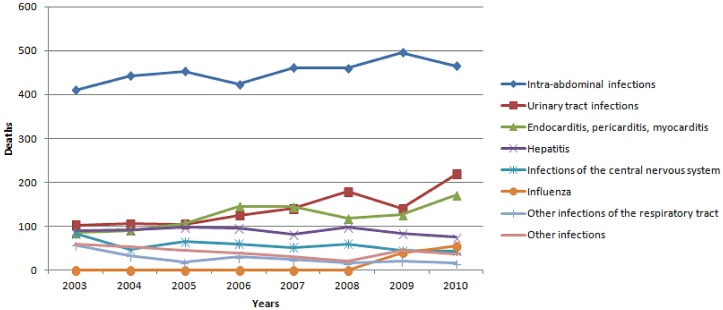
Deaths due to miscellaneous infections by year in Greece during 2003–2010.

Other infectious causes of death were distributed as follows: 31,904 (70.2%) due to pneumonia, 661 (1.5%) due to pulmonary TB, 96 (0.2%) due to influenza, 216 (0.5%) due to other infections of the respiratory tract, 3,611 (7.9%) due to IAIs, 1,116 (2.5%) due to UTIs, 989 (2.2%) due to endocarditis or pericarditis or myocarditis, 718 (1.6%) due to hepatitis, 455 (1%) due to CNS infections, and 332 (0.7%) due to other infections. Table 1 shows the percentage of deaths by site of infection each year. There were more deaths due to pneumonia, IAIs and UTIs during 2007–2010 than 2003–2006 [17,750 versus 14,154 (20.3% increase); 1,882 versus 1,729 (8.1% increase); 678 versus 438 (35.4% increase) respectively (p<0.05 for all comparisons)]. Figure 2 and 3 shows the temporal trend of mortality due to pneumonia and pulmonary TB, and other infections respectively during the study period.

**Table 1:** Deaths caused by infectious diseases in Greece from 2003 to 2010 stratified by site of infection.

Infection	Deaths, n (%)								All years
	2003	2004	2005	2006	2007	2008	2009	2010	
Septicemia	471 (10.1)*	419 (8.8)*	514 (9.6)*	580 (11.3)*	760 (12.1)*	922 (14.6)*	992 (14.9)*	898 (14.3)*	5,556 (12.2)**
Pneumonia	3,199 (68.7)*	3,384 (71.4)*	3,884 (72.2)*	3,557 (69.3)*	4,469 (71.4)*	4,379 (69.1)*	4,590 (68.9)*	4,239 (67.4)*	31,701 (69.7)**
Pulmonary TB	101 (2.2)*	69 (1.5)*	86 (1.6)*	78 (1.5)*	93 (1.5)*	80 (1.3)*	84 (1.3)*	70 (1.1)*	661 (1.5)**
Influenza	0 (0)*	0 (0)*	1 (0.02)*	0 (0)*	0 (0)*	0 (0)*	40 (0.6)*	55 (0.9)*	96 (0.2)**
Other RTIs	57 (1.2)*	34 (0.7)*	18 (0.3)*	30 (0.6)*	24 (0.4)*	16 (0.3)*	21 (0.3)*	16 (0.3)*	216 (0.5)**
IAIs	410 (8.8)*	443 (9.3)*	453 (8.4)*	423 (8.2)*	461 (7.4)*	460 (7.3)*	496 (7.4)*	465 (7.4)*	3,611 (7.9)**
UTIs	103 (2.2)*	106 (2.2)*	104 (1.9)*	125 (2.4)*	140 (2.2)*	179 (2.8)*	140 (2.1)*	219 (3.5)*	1,116 (2.5)**
Endocarditis, pericarditis, myocarditis	85 (1.8)*	91 (1.9)*	107 (2)*	146 (2.8)*	145 (2.3)*	118 (1.9)*	126 (1.9)*	171 (2.7)*	989 (2.2)**
Hepatitis	91 (2)*	92 (1.9)*	98 (1.8)*	97 (1.9)*	82 (1.3)*	99 (1.6)*	84 (1.3)*	75 (1.2)*	718 (1.6)**
CNS infections	83 (1.8)*	47 (1)*	65 (1.2)*	60 (1.2)*	52 (0.8)*	60 (0.9)*	45 (0.7)*	43 (0.7)*	455 (1)**
Other infections	59 (1.3)*	53 (1.1)*	46 (0.9)*	40 (0.8)*	30 (0.5)*	21 (0.3)*	45 (0.7)*	38 (0.6)*	332 (0.7)**
**Total**	**4,659**	**4,738**	**5,376**	**5,136**	**6,256**	**6,334**	**6,663**	**6,289**	**45,451**

* The percentage is calculated using the total infections of each year. ** The percentage is calculated using the total infections of each site during the whole study period. **Abbreviations:** TB: tuberculosis, RTI: respiratory tract infection, IAI: intra-abdominal infection, UTI: urinary tract infection, CNS: central nervous system.

## Discussion

Data from the Hellenic Statistical Authority indicates that septicemia was ranked at the second position among the infectious causes of death after pneumonia in Greece during the study period. More deaths were attributed to septicemia at the second quadrennial (2007–1010) of the study period. This data is consistent with the respective in the USA where pneumonia caused most deaths among infections in 2011 and was ranked at the 8^th^ position among all causes of death, while septicemia was the second most fatal infection ranked at the 11^th^ position. [1].

Interestingly, the causative pathogen of septicemia was not reported in the vast majority of deaths, partially because clinicians might consider as priority to report the disease which caused the death rather than the pathogen which caused the disease on the death certificate. Since data on septicemia due to *Staphylococcus* spp, *Streptococcus* spp and anaerobes was recorded, it could be assumed that indeterminate septicemia was probably caused by Gram-negative bacteria or fungi or pathogens that were not identified. As it was previously stated, Greece is facing a serious problem with multidrug-resistant infections, mainly caused by Gram-negative pathogens. Many outbreaks have been recorded across the Greek territory caused by carbapenem-resistant Enterobacteriaceae [6], [7], [8], [9], [10], [11] and carbapenem-resistant non-fermentative bacilli [12], [13], [14] or even Enterobactericaeae producing extended-spectrum beta-lactamases. [15], [16] It has been shown that the problem of multidrug resistance is greater in the intensive care units where patients usually have high co-morbidity. [17], [18] Nevertheless, infections caused by multidrug- resistant pathogens are recorded in the community in Greece, as well. [19], [20] Nosocomial or community-acquired infections due to multidrug-resistant pathogens may lead to septicemia more easily in patients with co-morbidities and impairment of functional health status than in patients with no underlying disease. Accordingly, the high prevalence of multidrug-resistant infections in Greece during the last years may account for the continuous increase in deaths due to septicemia which is overall characterized, by high mortality. [21].

Apart from septicemia, it is shown that deaths due to pneumonia, IAIs, and UTIs significantly increased after 2007 in Greece, as well. This finding could partially be justified by the spread of multidrug-resistant pathogens across the country which causes difficult-to-treat nosocomial infections. At this point, it should be addressed that according to data from the National Action Plan for multidrug-resistant Gram-negative infections called “Procrustes”, which was first applied in November 2010, [22] pneumonia was the infection which caused most deaths (44.9%) during the first semester of 2011 in Greece followed by bacteremia (40%). [23] In addition, the same data indicates that bacteremia had the highest prevalence among nosocomial infections (34.8%) followed by pneumonia (29.8%), UTIs (21%), and surgical site infections (14.4%).

A slight increase was also observed in the mortality rate due to endocarditis, pericarditis and myocarditis after 2009. Taking into consideration the pathogenesis of these infections according to which a viral infection is in most times the responsible factor, the outbreak of H1N1 infection in 2009 might be a reason for the increase in deaths due to endocarditis, pericarditis and myocarditis. With regard to H1N1 infection, the outbreak in 2009 is clearly identified with the increase in deaths caused by influenza after 2009. However, it should be highlighted that the lack of deaths due to influenza before 2009 seems not to reflect the reality, but, probably the fact that clinicians were less sensitized to the diagnosis of influenza before that outbreak and consequently, cases of influenza may have remained undiagnosed. In addition, it seems possible that a percentage of deaths caused by pneumonia may in fact represent secondary pneumonia after influenza.

In addition, it is worthwhile mentioning that the mortality rate due to pulmonary TB, CNS infections and hepatitis, exhibits stability over time. These infections can partially be prevented through vaccination and our results denote high adherence of the population to the national immunization program. In fact, data from Hellenic Centre for Disease Control and Prevention indicates that the prevalence of TB, meningitis and hepatitis has a downward trend during the last decade, [24], [25], [26] despite the increased influx of immigrants in Greece from countries with high prevalence of the aforementioned infectious diseases.

Since the high prevalence of multidrug-resistant pathogens in Greece results in increasing mortality by causing septicemia, the strengthening of infection control measures in the hospital facilities is now essential. Besides the high mortality rate which is the most important reason, another reason that could also alarm the managers of the healthcare systems is that septicemia is a common and costly infection. In 2009, septicemia was the 6^th^ most common and the most expensive (nearly $15.4 billion in total hospital costs) reason for hospitalization in the USA. [27] Accordingly, early laboratory detection of the multidrug-resistant strains and surveillance cultures for the detection of colonized patients are among the major preventive measures that should be enhanced. In addition, isolation precautions for patients carrying such strains as well as dedicated staff for their treatment are necessary. Finally, antibiotic stewardship programs applied both in the hospital and the community would be another effective measure, since prior use of certain antibiotics is associated with the development of infections due to resistant pathogens. [9], [28], [29], [30], [31].

Despite the limitations arising from the quality of death certificates, this study shows that the mortality rate due to septicemia has almost doubled after 2007 in Greece. Proportionally, septicemia accounted for a greater increase in the mortality rate within the infectious causes of death. Infectious causes of death having the greatest increase among deaths caused by infectious diseases. The competent authorities should urgently act upon this serious problem which tends to become an epidemic by implementing stricter measures in the healthcare facilities across the Greek territory.
